# Impact of tele-exercise on quality of life, physical fitness, functional capacity and strength in different adult populations: a systematic review of clinical trials

**DOI:** 10.3389/fspor.2025.1505826

**Published:** 2025-01-30

**Authors:** Nuno Domingos Garrido, Victor Machado Reis, José Manuel Vilaça-Alves, Gabriela Chaves Lucas, Ismael Lima Godinho, Rafael Peixoto, Alberto Fucarino, Manuela Cantoia, Enzo Iuliano, Andrea De Giorgio, Antonio Fabbrizio, Martina Suasa, Giovanna Zimatore, Carlo Baldari, Filippo Macaluso

**Affiliations:** ^1^ Research Center in Sports Sciences, Health Sciences and Human Development (CIDESD), Vila Real, Portugal; ^2^ Sports Science, Exercise and Health Department, University of Trás-os-Montes & Alto Douro, Vila Real, Portugal; ^3^ Department of Theoretical and Applied Sciences, eCampus University, Novedrate, Italy; ^4^ Department of Biomedicine, Neuroscience and Advanced Diagnostics (BIND), University of Palermo, Palermo, Italy

**Keywords:** physical exercise, elderly, technology, health, tele-exercise

## Abstract

**Introduction:**

This study aimed to review the impact of tele-exercise on different adult populations, comparing synchronous and asynchronous interventions and their effects on outcomes such as quality of life, physical fitness, functional capacity, strength, and pain.

**Methods:**

Randomised clinical trials and quasi-experimental studies published between 2014 and 2024 were included, totalling 16 studies with 1,416 participants. The interventions varied between synchronous teleexercise (via videoconference) and asynchronous (via apps and recorded videos). The review followed the PRISMA guidelines, ensuring a systematic approach to study selection, data extraction, and bias assessment.

**Results:**

The results indicate that tele-exercise, especially synchronous, has the potential to primarily improve physical fitness, functional capacity, and pain perception, being effective for the elderly and individuals with specific conditions such as multiple sclerosis and obesity.

**Discussion:**

However, the methodological heterogeneity of the studies and the lack of consistent data limit the generalisation of the findings, highlighting the need for more high-quality research.

**Systematic Review Registration:**

https://www.crd.york.ac.uk/prospero/display_record.php?ID=CRD42024563241, PROSPERO (CRD42024563241).

## 1 Introduction

Recent advances in technology and artificial intelligence, along with the COVID-19 era, have driven the development and popularisation of various online telehealth tools (1). Telehealth, including the adaptable concept of tele-exercise, has emerged to meet the need to incorporate physical exercise into current demands, empowering individuals to take control of their health (2, 3).

Physical exercise can be defined as a structured activity aimed at improving and maintaining physical fitness (4). Tele-exercise, in turn, is an online exercise prescription methodology that can be performed synchronously or asynchronously. Among the tools that comprise tele-exercise are training apps for smartphones with personalised programs, synchronous training via videoconference or mobile calls, apps with recorded videos to guide into workouts, activity monitoring via smartwatches and smart homes, and virtual assistants that suggest and monitor activities (2, 3, 5, 6). The advancement of these tools can be a resource for exercise training and adherence for individuals with mobility restrictions, health issues, and even people with limited access to in-person exercise environments, such as many elderly or individuals with limitations and comorbidities (7–9).

Given the global trend of low physical activity levels, it is concerning that 31% of adults and 80% of adolescents do not meet the World Health Organization's recommendations for physical activity (10), representing a significant public health challenge.

Given the trend towards sedentary lifestyles, tele-exercise has been introduced as a potential tool for expanding physical exercise (9). A basic tele-exercise program consists of a central database using web services to connect healthcare providers to patients' devices virtually. With technological advancements, tele-exercise using high-speed internet, videoconferencing, and smartphone apps can allow individuals to engage in physical activities remotely under the supervision of experienced and trained professionals. Importantly, these tools can enable participants to choose their preferred exercise regimes, especially those requiring minimal supervision (6, 11), thereby catering to their individual fitness needs and making them feel considered in their fitness journey.

These regimes help maintain regularity while minimising costs. Webcams provide communication between healthcare professionals and are a method for monitoring patients' activity (12). As one of the most recent branches of telemedicine, the theoretical data on its effectiveness lacks sufficient practical data to support it.

During the COVID-19 pandemic, the importance of tele-exercise as a health alternative was put into perspective (1, 13). Although some systematic reviews have addressed the topic of tele-exercise, its role in health, particularly regarding improvements in quality of life, physical fitness, and sub-groups such as functional capacity and strength in adult populations, is not yet well defined. Thus, this study primarily aims to understand the impact of tele-exercise on different population groups. Secondarily, it seeks to compare synchronous vs. asynchronous tele-exercise and tele-exercise vs. in-person exercise, assessing whether there are differences in impact on quality of life, physical fitness, functional capacity, and strength across different adult populations based on the exercise method used. Therefore, our focus is primarily on randomised clinical trials, recognising the importance of systematic reviews that include such studies. However, acknowledging that the current literature on the topic is recent, this work also accepts quasi-experimental studies that meet the eligibility criteria to make the review more comprehensive.

## 2 Methods

### 2.1 Registration protocol

The protocol was registered with the International Prospective Register of Systematic Reviews—PROSPERO (registration number CRD42024563241) prior to the start of this review, which was drafted in accordance with the PRISMA 2020 statement (14).

### 2.2 Eligibility criteria

#### 2.2.1 Inclusion criteria

The selection process was meticulous and thorough. Initially, it was considered various study designs, but upon discovering a substantial number of clinical trials and quasi-experimental studies, it was chosen to focus on these two designs. Whether randomised or not, or included control groups, it was sought study designs that most effectively address the research question while rigorously accounting for their methodological limitations through stringent risk of bias criteria. Studies published between 2014 and 2024 with no language restrictions were included. The criteria was stringent, requiring at least one tele-exercise intervention, either synchronous or asynchronous, with a minimum duration of 6 weeks.

The present review encompasses a diverse range of adult populations. Adults aged between 18 and 99 who were not hospitalised or undergoing cardiac rehabilitation or physiotherapy programs were included. There were no restrictions regarding the population, resulting in a rich heterogeneity. The population categories included elderly individuals, elderly individuals in cancer remission, pregnant women with obesity or overweight, obese adults, adults with Parkinson, adults with multiple sclerosis (MS), adults with Down syndrome, adults with recent COVID-19, and healthy adults.

Accommodating a wide range of tele-exercise interventions, it were considered studies with synchronous and/or asynchronous tele-exercise interventions of any structured training method (e.g., resistance training or yoga). Synchronous interventions were required to be conducted via live videoconferencing, either in groups or individually, using smartphones, tablets, or computers. Asynchronous interventions could utilise technologies such as recorded videos, remote training apps, or traditional methods like prescription and supervision via phone calls. Asynchronous interventions could involve either one-time or ongoing interactions.

The main outcomes of this review included data assessing changes from baseline over time by group or between groups. Studies were included if they assessed at least one outcome related to physical fitness, quality of life, functional capacity, strength, and pain, evaluated using a validated assessment tool. The presence of a control condition was not a mandatory inclusion criterion.

Studies with synchronous and/or asynchronous tele-exercise interventions of any structured training method (e.g., resistance training or yoga) were included. However, synchronous interventions needed to be conducted via live videoconferencing, either in groups or individually, using smartphones, tablets, or computers. Asynchronous interventions could utilise technologies such as recorded videos, remote training apps, or traditional methods like prescription and supervision via phone calls. Asynchronous interventions could involve either one-time or ongoing interactions.

#### 2.2.2 Exclusion criteria

Studies were excluded if they addressed telemedicine or telerehabilitation in the context of physiotherapy or post-surgical recovery, as well as those related to virtual reality environments and e-sports. Studies without a structured tele-exercise program were also excluded. Articles published before 2014 were disregarded. Given the rapid technological advancements, we chose to prioritise more recent publications that reflect current technological tools.

### 2.3 Search strategy

On July 6, 2024, and July 10, 2024, searches were conducted in four databases: PUBMED (74 articles), SCOPUS (59 articles), WEB OF SCIENCE (54 articles), and SCIENCE DIRECT (53 articles), totalling initially 240 articles. The search terms used (Table 1) were “Tele-exercise,” “Remote exercise,” “Remote training,” “Tele-exercise programs,” “Synchronous exercise,” and “Asynchronous exercise,” separated by the boolean operator OR, with a filter for “Title/Abstract” and coverage from 2014 to the present. A “snowball” search was also performed in the reference lists of the full texts, identifying two studies of interest. The entire process was conducted independently by two investigators. Table 1 presents the search strategy used for PUBMED.

**Table 1:** Search strategy.

NUMBER OF SEARCH TERMS OR COMBINATIONS USED (PUBMED)
#1 “Synchronous exercise”
#2 “Asynchronous exercise”
#3 “Synchronous exercise”
#4 “Tele-exercise programs”
#5 “Remote training”
#6 “Remote exercise”
#7 Teleexercise
#8 Tele-exercise
#9 OR/1-8
#10 “Randomized Controlled Trial”
#11 “Clinical Trial”
#12 “Randomized Clinical Trial”
#13 “Randomised Controlled Trial”
#14 “Trial”
#15 “Clinical study”
#16 “Comparative study”
#17 “Observational study”
#18 “Cohort study”
#19 “Case-control study”
#20 “Cross-sectional study”
#19 “Prospective study”
#20 OR/10-19
#21 9 AND 20
FILTERS
NOT REVIEWS
FROM 2014 ON

#### 2.3.1 Permission to reuse and copyright

No permission was necessary for the use of copyrighted content.

### 2.4 Study selection process

**Figure 1:**
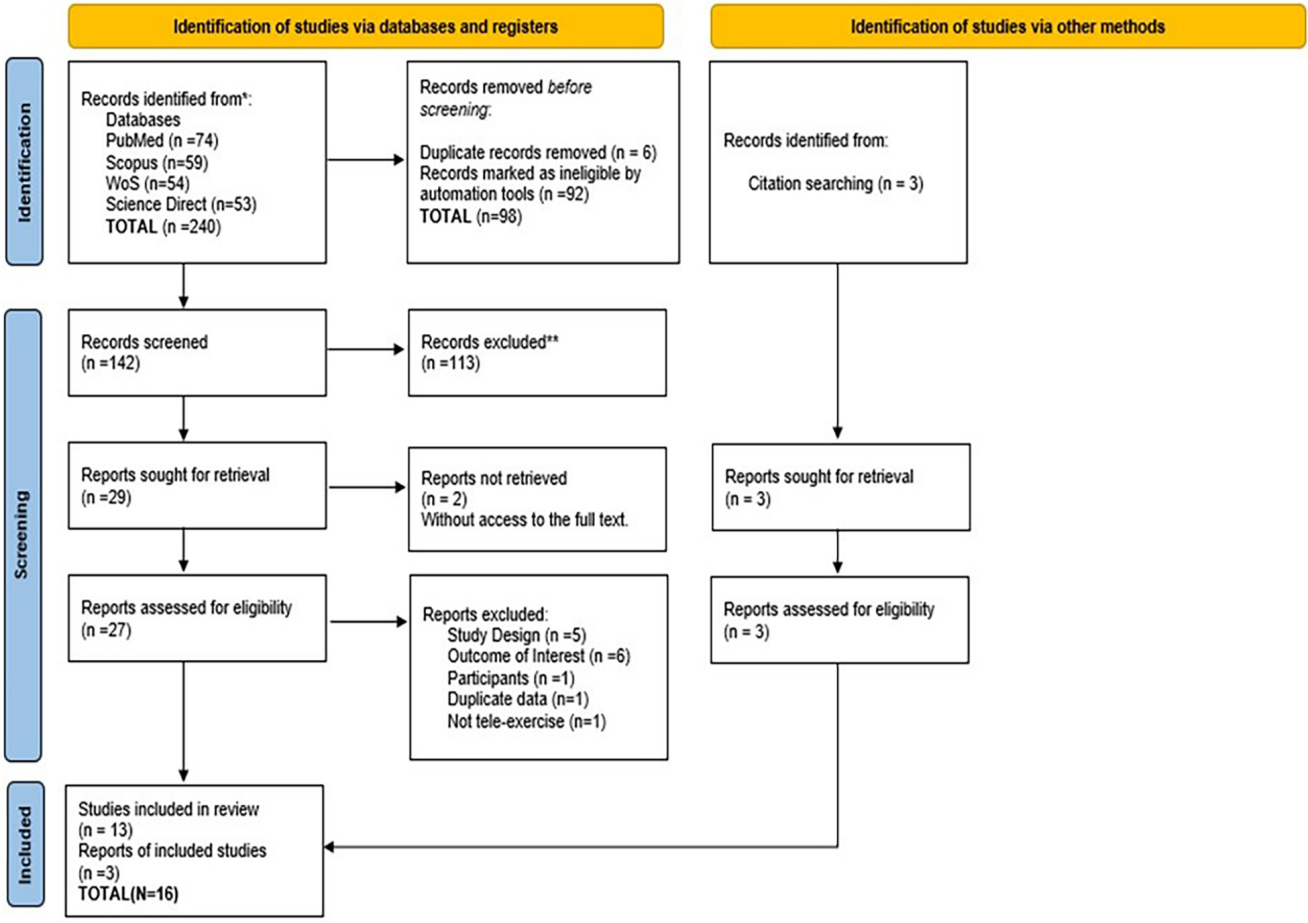
PRISMA flowchart outlining the flow of studies through the review.

The initial reference screening was conducted using the reference management software ZOTERO 6.6.27. A total of 240 studies were initially identified (the flow diagram is provided in Figure 1 in Section 3). Duplicate records were identified using software, reviewed manually, and removed accordingly. Two reviewers independently assessed the “Title and Abstract,” and any discrepancies were discussed until a consensus was reached. The same reviewers applied the inclusion and exclusion criteria during the full-text selection process and cross-checked the results. Discrepancies were resolved by consensus without needing a third reviewer (more details in Section 3.1).

### 2.5 Data extraction

After applying all exclusion criteria and finalising the list of studies, data from each study were extracted by two independent reviewers using a data extraction sheet adapted from the Critical Appraisal Skills Programme (CASP) for randomised clinical trials. Two other reviewers focused on extracting general data and study characteristics for a descriptive assessment of each study (Table 2). Discrepancies were resolved through discussion or with the assistance of a third reviewer.

**Table 2:** Study characteristics.

Study	Study Desing	Intervention Type	Population	Outcome	Results
Ayse et al., (15)	Randomised clinical trial	6 weeks of tele-exercise synchronous IG vs. control	Isolated elderly (*n* = 30)	Physical Fitness (SFT) Quality of life (NHP)	IG × CG: There was a significant improvement in all outcomes of the Senior Fitness Test (SFT), including strength and quality of life.
Bernardo et al., (7)	Randomised Clinical trial	8 weeks of tele-exercise asynchronous IG vs. CG (control)	Obese and overweight pregnant woman (*n* = 24)	Physical fitness (PPAQ) Pain (ODI)	IG × CG: There was a significant difference in light and moderate physical activity levels, and a reduction in pain markers.
Buckinx et al., (26)	Non-randomised clinical trial	12 weeks of tele-exercise synchronous and asyncrhronous versys control.	Pre-independent elderly (*n* = 72)	Physical fitness (SPPB) and Tapa 1 e 2 Functional Status (OARS)	IG × CG: There were no significant differences between groups for any outcome.
					Moment × Group: The intervention group showed significant improvements in all outcomes.
Canton-martinez et al., (16)	Non-randomised clinical trial	6 weeks of tele-exercise synchronous IG vs. Control	Isolated elderly (*n* = 30)	Physical fitness (SFT)	IG × CG: There was a significant difference between groups for some of the SFT tests, namely sit-to-stand, 4-meter walk, TUG, and arm curl.
Da silva et al., (17)	Randomised clinical trial	8 weeks of synchronous group tele-exercise for both groups. The IG received nutrition coaching.	Obese and overweight women (*n* = 31)	Physical fitness (VO2)	IG × CG: There were no significant differences between groups.
					Group × Moment: Both groups showed significant improvements from baseline to the last week of intervention.
Ellis et al., (18)	Randomised clinical trial	12 months of asynchronous tele-exercise vs. an active control group	Adults with Parkinson's disease (*n* = 51)	Physical activity level (Pedometer) Quality of life (PDQ-39) Functional capacity (6-minute walk test, TC6)	IG × CG: There were no significant differences between groups; however, the “sedentary” subgroup showed significant improvements in quality of life and functional capacity.
Eyuboglu et al., (19)	Randomised clinical trial	Intervention group (IG) received 12 weeks of tele-yoga, while the control group (CG) engaged in chest expansion exercises.	Adults with sleep apnea syndrome. (*n* = 44)	Physical fitness (VO2máx) Functional capacity (TC6)	Group vs. Control Group: No significant difference between groups.
					Group × Moment: Significant difference was observed only for functional capacity.
Ferrari et al., (8)	Randomised clinical trial	6 months of tele-exercise asynchronous vs. control	Healthy elderly (*n* = 37)	Balance (30 s of static balance), Functional capacity (10TC e 5-sit to stand)	IG × CG: No significant difference was observed for any of the outcomes between the groups.
					Group × Moment: The intervention group showed significant improvement in functional capacity and strength over time.
Gell et al., (25)	Randomised clinical trial	16 weeks of synchronous tele-exercise vs. control.	Older adults in remission from different types of cancer. (*n* = 37)	Physical fitness (Level of de PA), quality of life (PROMIS-29), balance and strength (Sit to Stand).	IG × CG: There was a significant difference between groups only for physical activity level.
					Group × Moment: Over time, there was a significant difference for the outcomes of strength and quality of life.
Guerrero et al., (27)	Quasi-experimental	12 weeks of synchronous tele-exercise.	Down syndrome adults (*n* = 18)	Balance (FICSIT-4), agility/coordination (TUG) and strength (5-sit to stand e 30s CS)	There was a significant difference for all outcomes.
Hong et al., (20)	Randomised clinical trial	The IG participated in 12 weeks of synchronous tele-exercise vs. a control group.	Elderly (*n* = 23)	Physical fitness (SFT)	IG × CG: There was no significant difference between groups for physical fitness in the SFT tests.
					Group × Moment: There was a time effect for some specific tests within the SFT, including the 2-minute steps, Sit and Reach, and Sit-to-Stand tests.
Jones et al., (21)	Quasi-experimental	16 weeks of synchronous tele-exercise Tai-Chi	Elderly (*n* = 52)	Balance (FSST), Agility/Coordination (TUG), Strength (5-Sit to Stand), and Functional Capacity (TC5 m)	There was a significant difference for all outcomes.
Najafi et al., (22)	Randomised clinical trial	IG 8 weeks of tele-yoga and tele-exercise vs. control	Adults with Multiple sclerosis (*n* = 82)	Physical fitness, quality of life and pain (ovQV), functional capacity (T25FW).	IG × CG: There were significant differences for all outcomes.
Najafi et al²., (23)	Randomised clinical trial	IG 8 weeks of tele-yoga and tele-pilates vs.	Women with Multiple Sclerosis (*n* = 45)	Physical fitness level (IPAQ), Quality of life (MSQol-54), and Functional capacity (T25FW)	IG × CG: There was a significant difference between groups for all outcomes.
Poon et al., (28)	Randomised clinical trial	The intervention group underwent 8 weeks of synchronous and asynchronous tele-exercise vs. control.	Adults with recent COVID infection. (*n* = 41)	Physical fitness (treadmil test), Quality of life (HRQol), Strength (Handgrip e Sit to Stand)	IG × CG: There was a significant difference in quality of life between the groups.
					Group × Moment: There was a significant group-time effect for the outcomes in strength (handgrip e sit to stand)
Wilke et al., (6)	Randomised clinical trial	IG Participated in 8 weeks of tele-exercise, with 4 weeks of synchronous tele-exercise and 4 weeks of asynchronous tele-exercise vs. Control for 4 weeks and Asynchronous Control for the last 4 weeks.	Adultos saudáveis (*n* = 763)	Aptidão fisica (NPAQ-Short) e Dor (Chronic Pain Grade)	IG × CG (4s) There was a significant difference in quality of life in the intention-to-treat analysis and CAGE. For pain, there was a difference between groups only in the CAGE analysis.

IG, intervention group; CG, Control Group.

Data was collected on:

• Report: Author and year of publication.
• Study: Type of study and research question.
• Participants: Characteristics of the population.
• Intervention: Type, duration, and FITT characteristics whenever available.
• Research design or characteristics: Outcome of interest, methodology, results, and effect size.

### 2.6 Quality criteria of primary studies

**Figure 2:**
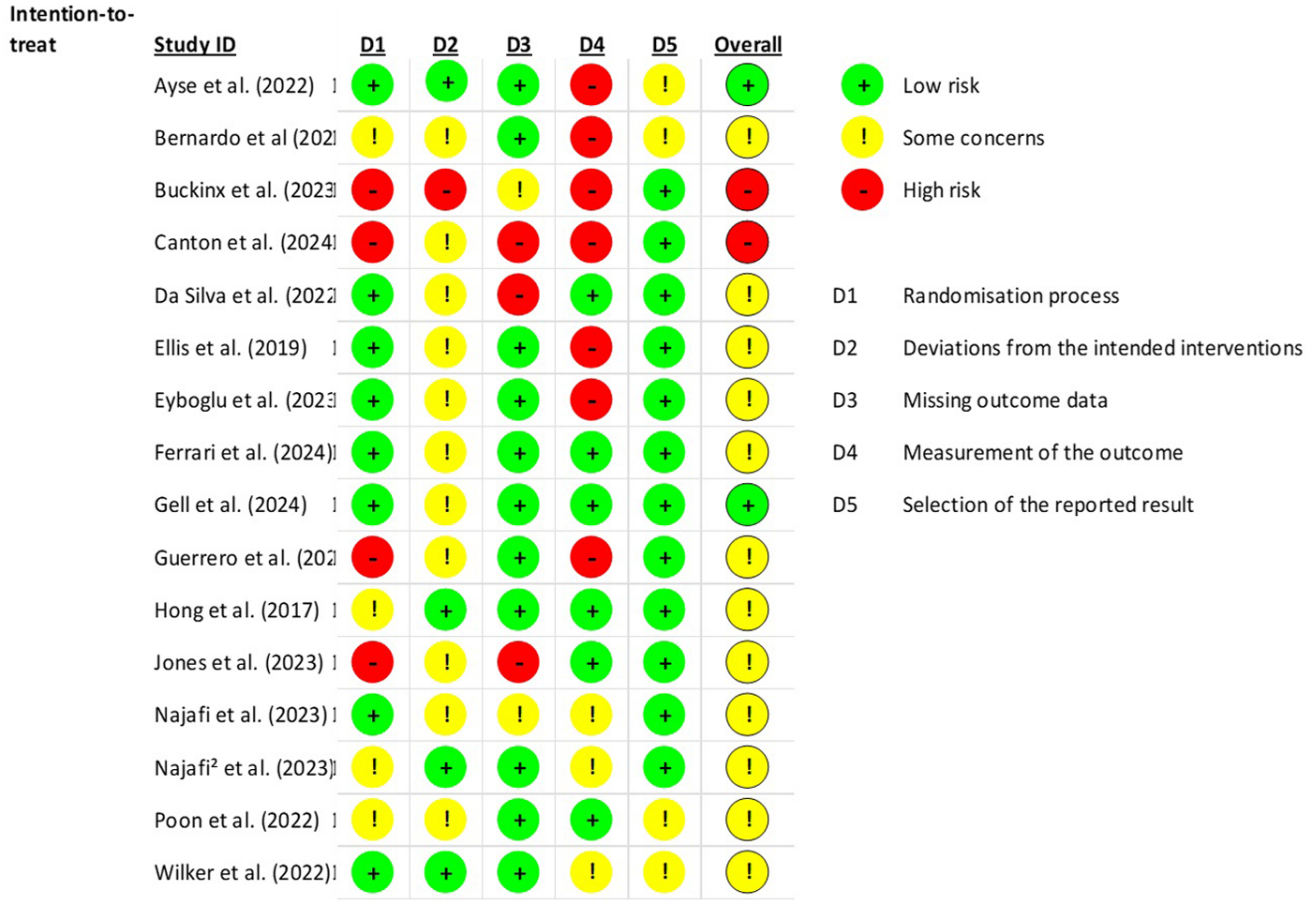
ROB.2 evaluation of included studies.

Two reviewers independently assessed the quality of the studies. In cases of disagreement that could not be resolved through mutual reconciliation, a third reviewer was consulted. The quality scale used was the ROB.2 Cochrane Risk-of-Bias Tool for Randomized Trials, given that randomised clinical trials are the primary focus of this study. The studies were assessed as having low, unclear, or high risk of bias (Figures 1, 2).

**Figure 3:**
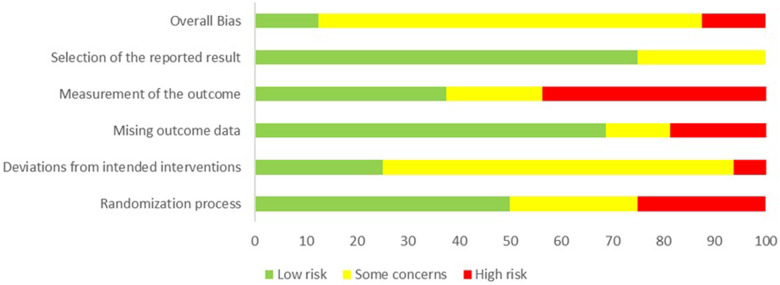
Risk of bias assessment with ROB.2 as percentage.

The risk of bias was assessed across five key domains: the randomization process (D1), deviations from the intended interventions (D2), missing outcome data (D3), measurement of the outcome (D4), and selection of the reported results (D5). Each domain includes subdomains designed to provide a detailed scope of each main domain.

### 2.7 Effect measures and data synthesis

The studies included in this systematic review exhibit heterogeneous samples, instruments, designs, and interventions, preventing the statistical precision required for a meta-analysis. Due to the heterogeneity in intervention methods and reported outcomes for different populations, we chose to present the data in tabular format, highlighting the characteristics of the interventions, population, and effect size of each article. Additionally, we summarise and present the data in a narrative synthesis in the text, by outcome in population subgroups, detailing the main characteristics of each article, results for all outcomes, and statistical significance.

The results were organised into main categories: quality of life, physical fitness, functional capacity, and strength. The secondary outcome of pain was also described within the main category of quality of life.

## 3 Results

### 3.1 Study selection

As mentioned earlier, a total of 240 studies were initially identified during the first screening in the databases. Ninety-eight duplicates were identified and removed. The “Title and Abstract” of 142 studies were examined, and 29 were selected for full-text screening. The full text of two studies could not be accessed. An email was sent to the corresponding authors, but no response was received. Of the 27 analysed studies, 5 were excluded for not being randomised or quasi-experimental trials, 6 did not have outcomes of interest, 1 did not involve an adult population, and 1 was considered not to have characteristics of structured tele-exercise. The study conducted by Jones in 2023 (24) reported results included in his other study published in 2024 (21). Only this latest was included and will be cited. Thus, 14 studies were excluded. From the full-text article list, 3 new studies were found (snowball effect). In total, we screened 242 studies, resulting in 16 eligible studies.

### 3.2 Characteristics of the studies

#### 3.2.1 Tele-exercise approach

Eleven of the sixteen studies were randomised clinical trials (6–8, 15, 17–20, 22, 23, 25). Five were non-randomised clinical trials or quasi-experimental studies. Two studies used a pragmatic approach to participant allocation due to restrictions during the COVID-19 pandemic (16, 26). Two studies were quasi-experimental with a single-group pre-post design (21, 27), and the fourth study did not report a specific reason for not being randomised (28).

Three studies presented the exclusively asynchronous approach. In the study by Ferrari et al. (2024), wearable technology with inertial sensors was used, featuring an interface that allowed the trainer to remotely prescribe workouts and provide movement feedback to the trainer. Participants received workouts via the web, including videos and demonstrations. Ellis et al. (2019) used a model based on remote monitoring, where all participants received tablets with personalised workouts.

The synchronous approach studies used various videoconferencing platforms such as Skype, Zoom, and Google Meet. All of them were conducted as group training sessions, with workouts ranging from tele-Pilates, tele-Yoga, and tele-Tai Chi to exercises incorporating strength, endurance, aerobic, flexibility, and balance components (7, 15–17, 19–23, 25, 27).

#### 3.2.2 Population

The population of the eligible studies was heterogeneous, totalling 1,416 participants. Six studies focused exclusively on the elderly. One study evaluated pre-dependent elderly individuals, four studies assessed healthy elderly individuals, one study evaluated elderly individuals with sub-groups for comorbidities (hypertension and diabetes), and one study evaluated elderly individuals in remission from various types of cancer (8, 15, 16, 20, 21, 25, 26). Ellis et al. (2019) assessed adults and elderly individuals with Parkinson's disease, with the majority being elderly (18). Two studies focused on individuals with obesity and overweight, with one study including pregnant women (7) and the other including the general obese population (17). Four studies examined the adult population, including adults with Down syndrome (27), adults with sleep apnea syndrome (19), adults with recent COVID-19 (28), and healthy adults (6).

A summary of the characteristics, intervention types and outcomes is shown in Table 2.

### 3.3 Quality of evidence and strength of recommendations

Two researchers independently assessed the evidence using GRADE (Grading of Recommendations, Assessment, Development and Evaluation) criteria (29) with the assistance of the GRADE PRO GDT software, available at http://gdt.gradepro.org/. The quality of evidence and strength of recommendations can be seen below (Table 3).

**Table 3:** Outcomes grades of evidence.

Summary of results:			
Tele-exercise Versus Control for Different Outcomes			
Population: Different adult populations including elderly people			
Intervention: Tele-exercise synchronous and Asynchronous			
Comparison: Control/Pre-Post			
Outcomes	Impact	Participants	Certainty of the evidence
		(Study design)	(GRADE)
Physical Fitness	Although the outcome physical fitness was affected by the downgrade, it still holds a moderate level of recommendation, especially for key target populations such as the elderly and healthy adults. The moderate rating indicates that, despite some uncertainty regarding the exact magnitude of the benefits, there is sufficient evidence to support tele-exercise interventions aimed at improving physical fitness in these populations. The strength of the recommendation is high, reflecting the potential impact of these interventions despite some methodological limitations and heterogeneity in the studies.	1,416 (11 ECRs) (2 not Randomised) (2 Quasi-experimental)	⊕⊕⊕○ Moderate^a^
Quality of Life	The downgrading of the quality of life outcome was necessary due to the heterogeneous nature of the studied populations and the low methodological quality of some studies. Therefore, we consider the strength of the recommendation to be low.	245 (5 ECRs) (1 Quasi-experimental)	⊕⊕○○ Low^a^^,^^b^
Functional Capacity	Although the outcome functional capacity was impacted by the downgrade, it still maintains an important level of recommendation, especially for specific target populations such as the elderly and healthy adults. The moderate rating suggests that, while there is some uncertainty regarding the exact magnitude of the benefits, there is sufficient evidence to support tele-exercise interventions aimed at improving functional capacity in these populations. The strength of the recommendation is high.	365 (5 ECRs)	⊕⊕⊕○ Moderate^a^
Strength	The downgrading of the strength outcome was necessary mainly due to the small sample size, the nature of the tests used, and the methodological quality of some studies.	127 (3 ECRs) (1 not randomized)	⊕⊕⊕○ Moderate^a^^,^^b^^,^^c^
Pain	Although the outcome of pain was affected by the downgrade, it still holds a moderate level of recommendation, especially for the adult population and in the context of synchronous tele-exercise. The moderate rating indicates that, despite some uncertainty regarding the exact magnitude of the benefits, there is sufficient evidence to support tele-exercise interventions aimed at improving pain, with a high strength of recommendation.	811 (3 ECRs)	⊕⊕⊕○ Moderate^d^
GRADE Working Group grades of evidence			
High certainty: we are very confident that the true effect lies close to that of the estimate of the effect.			
Moderate certainty: we are moderately confident in the effect estimate: the true effect is likely to be close to the estimate of the effect, but there is a possibility that it is substantially different.			
Low certainty: our confidence in the effect estimate is limited: the true effect may be substantially different from the estimate of the effect.			
Very low certainty: we have very little confidence in the effect estimate: the true effect is likely to be substantially different from the estimate of effect.			

Explanations.

^a^ Due to the heterogeneity of the population and the different protocols used for tele-exercise and assessment.

^b^ Due to the low methodological quality found in some studies.

^c^ Reduced number of studies.

^d^ Failure in blinding of the assessor and other risks of bias.

### 3.4 Results by outcomes

Different assessment tools, training methodologies, and statistical models were used to present the results of the eligible studies. In this section, we will synthesize these results by outcomes, specifying the population of each study and maintaining the statistical data in their original scales.

#### 3.4.1 Physical fitness

We considered physical fitness outcomes related to the level of physical activity and/or aspects associated with physical capacity, except for strength, which will be presented separately.

All eligible studies reported results for at least one physical fitness marker. The most used marker was the level of physical activity (6, 7, 18, 22, 23, 25, 26). Bernardo et al. (2024) evaluated obese and overweight pregnant women using the Pregnancy Physical Activity Questionnaire (PPAQ) and reported significant differences for increases in light and moderate physical activity, with a moderate effect size (*p* = 0.025, ES = 0.50; *p* = 0.005, ES = 0.66). No significant difference was found in the level of vigorous physical activity.

A study evaluated the level of physical activity in the elderly. It assessed pre-dependent elderly individuals using the Telephone Assessment of Physical Activity (TAPA 1 and 2), finding no significant differences between groups (26). However, there was a moment effect for the intervention group on TAPA 1 and 2 (*p* = 0.06; *p* = 0.007), although the effect size (ES) for each outcome was not reported. In another study, the authors objectively evaluated the physical activity level of elderly cancer survivors using an accelerometer (25). There was a significant difference with an increase in light and moderate physical activity, accompanied by a significant increase in the number of daily steps, with large effect sizes in the intervention group (respectively, *p* = 0.03, ES = 0.72; *p* = 0.001, ES = 0.81; *p* = 0.01, ES = 0.96).

Adults with Parkinson's disease were evaluated for physical activity levels using a pedometer to measure step count. Still, no significant differences were found between groups, nor was there a moment effect (18). Two studies assessed physical activity levels in patients with Multiple Sclerosis, both subjectively, using the International Physical Activity Questionnaire (IPAQ). For adults with Multiple Sclerosis, there was a significant difference between groups for moderate physical activity (*p* = 0.01, *η*² = 0.20), and for all activity levels, there was a moment effect with small effect sizes (23). In women with Multiple Sclerosis, two distinct tele-exercise models, tele-pilates (*p* = 0.002, *η*² = 0.52) and tele-yoga (*p* < 0.001, *η*² = 0.68), showed significant differences between groups when compared to the control group.

One study assessed physical activity levels in healthy adults. Wilke et al. (2022) subjectively evaluated the levels using the Nordic Physical Activity Questionnaire-short (NPAQ) after eight weeks of tele-exercise, with four weeks of synchronous and four weeks of asynchronous sessions, measured at eight time points. The greatest gains occurred during the first four weeks of synchronous tele-exercise vs. the control group, with increases of 1.65 and 1.39 in moderate and light physical activity, respectively (6).

Two physical fitness batteries were also used to assess older adults. Buckinx et al. (2021) used the Short Physical Performance Battery (SPPB) (26). Although no group effect was observed, there was a significant moment effect in the intervention group, reflected in the increase in total SPPB score (*p* = 0.004). Hong et al. (2017) used the Senior Fitness Test (SFT), with only flexibility showing significant differences between groups (*p* = 0.019). For the moment effect, the 2 min-steps cardiorespiratory fitness test showed a significant improvement in the intervention group (*p* = 0.011). In the study conducted by Ayse et al. (2022), the authors identified significant differences in three SFT tests between groups: 2 min step test (*p* ≤ 0.001), Sit and Reach Test (*p* = 0.028), and 8 Step Up and Walk Test (*p* = 0.010). Additionally, significant differences were reported between groups in the SFT for the 4-meter Gait Speed (*p* = 0.011, *η*_p_² = 0.20) and 8 Foot Up and Walk (*p* = 0.018, *η*_p_² = 0.18) tests, with small effect sizes (15).

Three studies specifically evaluated balance. Ferrari et al. (2024) used the 30-second static balance test to assess older adults, but no significant differences were found between groups, nor was there a moment effect (8). However, using the Four-Square Step Test, Jones et al. (2024) observed a moment effect (*p* = 0.45, *d* = −0.37) (21). A moment effect was also observed (*p* = 0.019, ES = 0.569) in the balance of adults with Down syndrome, assessed with the Four Stage Balance Test (FICSIT-4) (27). The Timed Up and Go test, commonly used to assess balance and agility in adults with mobility limitations, was used in two studies. In the study mentioned before, by evaluating older adults, Jones et al. (21) did not observe significant differences. However, in adults with Down syndrome, a moment effect was observed (*p* = 0.043, ES = 0.569) (27).

Only three studies specifically assessed cardiorespiratory capacity. One study was conducted with overweight and obese women (17), another with adults with sleep apnea (19), and the last with adults who had recently had COVID-19 (28). No significant group effects were observed in any of the populations. However, for peak VO2, a significant difference with a small group-moment effect size was observed in overweight and obese women (*p* = 0.007, ES = 0.36) (17).

#### 3.4.2 Quality of life

Six studies assessed quality of life using different tools. Ellis et al. (2019) used the Parkinson Disease Questionnaire 39 (PDQ-39) to evaluate the effect of tele-exercise on the quality of life in people with Parkinson's disease, finding no significant differences between groups. However, a significant improvement was observed in the subgroup of sedentary individuals (*p* = 0.03). In older adults in cancer remission, assessed with the Patient-Reported Outcomes Measurement Information System-29 (PROMIS), a moment effect was observed (*p* = 0.04, *d* = 0.33) (25).

Two studies evaluated individuals with Multiple Sclerosis using two different instruments: Overall Quality of Life and Health (ovQV), which assesses general quality of life, and Multiple Sclerosis Quality of Life-54 (MSQoL-54), specific to multiple sclerosis. Both instruments showed significant improvement with group effects (*p* = 0.01, *η*² = 0.12; *p* < 0.0001) respectively (22, 23).

Poon et al. (2024) used the Health-Related Quality of Life (HRQoL) scale to assess adults with recent COVID-19 and observed a significant difference between groups (*p* = 0.04). The last instrument used was the Nottingham Health Profile (NHP) to evaluate the quality of life related to health in isolated older adults, demonstrating significant improvement for this population (*p* = 0.011) (15).

#### 3.4.3 Functional capacity

Three studies used the 6-Minute Walk Test (6MWT). Canton-Martínez et al. (2024) observed a significant improvement in the distance covered by the elderly in the intervention group (*p* ≤ 0.0001; *ηp*² = 0.39) (16). In adults with Parkinson's disease (18), only a moment effect was observed (*p* = 0.02), as well as in adults with sleep apnea syndrome (*p* = 0.003) (19).

Two other objective tests were used to evaluate older adults. Ferrari et al. (2024) used the 10-meter Walk Test and found no significant difference between groups; however, a moment effect with a moderate effect size was observed (*p* < 0.001, ES = 0.59) (8). For the 5-meter Walk Test, Jones et al. (2024) observed a moment effect (*p* = 0.02, *d* = 0.43) (21).

To assess functional capacity in women with multiple sclerosis, the Timed 25-Foot Walk Test was used, showing a significant difference between groups (*p* < 0.0001). Lastly, the Older Americans Resources and Services (OARS) functional status scale was used to evaluate functional capacity in pre-dependent elderly individuals. Although no significant difference was observed between groups, a moment effect was noted for the intervention group (*p* = 0.02) (26).

#### 3.4.4 Strength

Physical capacity in terms of strength was assessed by nine studies, with the sit-to-stand test in its various forms being the most used instrument. Only two studies found significant differences between groups, both evaluating elderly individuals: Ayse et al. (2022) and Canton-Martínez et al. (2024) (respectively *p* ≤ 0.001 and *p* ≤ 0.0001, *η*_p_² = 0.38) (15, 16). Six studies demonstrated a moment effect in the sit-to-stand test. In the sit-to-stand test, Gell et al. (2024) observed a moment effect in elderly individuals in remission from cancer (*p* < 0.0001) (25), Ferrari et al. (2024) in healthy elderly individuals (*p* = 0.009 ES = 0.26) (8), along with Hong et al. (2017) also in elderly individuals (*p* = 0.035) (20). Adults with recent COVID-19 also showed a moment effect (*p* = 0.003, *d* = 1.29) with a large effect size.

For the 5-sit to stand variation, a moment effect was observed in adults with Down syndrome with a moderate effect size (*p* = 0.014, ES = 0.55) (27), as well as in elderly individuals (*p* = 0.005, *d* = 0.51) (21). Upper limb strength was assessed in three studies. Obese and overweight women were evaluated using handgrip, and while there was no significant difference between groups, a moment effect with a small effect size was observed (*p* = 0.0006, ES = 0.25) (17). Similarly, adults with recent COVID-19 were also assessed using handgrip (*p* = 0.032, *d* = 0.50) (28). However, no group or moment effects were observed for arm curls in elderly individuals (20).

#### 3.4.5 Pain

Three studies assessed the pain outcome. Wilke et al. (2022) used the Chronic Pain Grade Scale (CPGS) and found no significant differences between groups of healthy adults (6). For adults with multiple sclerosis, a significant difference between groups was observed with a small effect size (*p* < 0.0001, *η*² = 0.22) (23). Obese and overweight pregnant women were evaluated using the Oswestry Disability Index for low back pain, which showed significant differences between groups with a large effect size (*p* = 0.001, ES = 0.82) (7).

## 4 Discussion

Physical fitness was the most studied outcome, with the level of physical activity being the most frequently used assessment tool. Synchronous tele-exercise appears to be an effective method for improving physical activity levels in different populations, showing significant group effects and moment effects (*p* < 0.05) in various studies (6, 7, 22, 23, 25). Interaction effects between groups were highlighted in studies involving adult, elderly, and multiple sclerosis populations, indicating a specific impact of the intervention on these populations (6, 22, 23, 25) However, the study by Ellis et al. (2019), which used an asynchronous tele-exercise model, did not show any effects on physical fitness between groups or over time (18). The reviewed studies feature highly diverse populations, and this was the only study focusing on adults with Parkinson's disease. Nonetheless, in Ellis's study, when dividing the training group into active and sedentary participant subgroups, the sedentary subgroup showed significant improvements in the studied outcomes. However, it is not possible to assert that these improvements are solely attributable to the intervention.

Recent studies have correlated the level of physical activity in individuals with Parkinson's disease with walking ability and the level of disease impairment, showing that moderate to vigorous physical activity appears to have more beneficial effects in reducing this impairment (29, 30). In the study in question, participants were asked to perform 5–7 exercises for a minimum of 3 days per week, although these could be done daily for a six-month period. However, there is no report on the perceived effort level or the total weekly training volume completed by each participant. Asynchronous long-term tele-exercise programs seem to have a lower capacity for maintaining training volume (6), and intensity markers, such as perceived exertion, can be used to regulate training intensity. It is possible that the training program met the volume and intensity needs of the sedentary group but fell short of the physical capacity of the active group. Studies with more robust designs are necessary to better clarify potential intervention effects for this specific population.

For obese and overweight pregnant women, the asynchronous protocol did not appear to have a negative influence, as significant group and moment effects were observed for improvements in light and moderate physical activity levels (7). This aligns with the current literature, where structured exercise is shown to improve overall physical activity levels in pregnant women (31). The study conducted by Bernardo et al. (2024) was short in duration, lasting eight weeks, and followed FITT (frequency, intensity, type, and time) guidelines. The intervention group consisted exclusively of nutritional modifications, which may have contributed to the absence of interaction effects, as both groups followed the same asynchronous intervention protocol.

Regarding overall physical fitness levels evaluated by two test batteries (SPPB and SFT), synchronous tele-exercise proved effective in improving general fitness components in elderly populations, particularly concerning moment effects. This finding aligns with current literature on physical exercise and fitness in this population (32–34). Only one study used an asynchronous protocol, but it employed a mixed methodology, where the same group underwent both synchronous and asynchronous protocols, making it impossible to separately evaluate the effects of each type of tele-exercise (26). However, in the study by Buckinx et al. (2021), the authors observed a moment effect across all SPPB tests, which suggests a similarity with the other results (26). It is important to highlight that the observation of the moment effect indicates a modification but not necessarily one specific to intervention. Further studies using similar test batteries are needed to better clarify this issue.

When evaluating general physical fitness in populations with Down syndrome, synchronous tele-exercise appears to provide significant improvements (27), aligning with current literature (35, 36). However, more robust research with control groups and larger sample sizes is necessary to clarify potential positive effects on the physical fitness of this population, as the only study addressing this group is quasi-experimental.

Physical fitness was also specifically assessed in terms of cardiorespiratory improvement. Only synchronous tele-exercise appears to generate significant effects on peak VO2, which was observed only in the sedentary population of obese and overweight women (17). Other populations evaluated for the same outcome did not show significant differences, even when the protocol involved synchronous tele-exercise (19, 28). This result may be attributed to the characteristics of the study population, obese and overweight women who had not previously engaged in physical activity, making them more responsive to the short-term beneficial effects on cardiorespiratory capacity. The different training protocols could also influence the outcome. Similarly, the study by da Silva et al. (2022) was unique among the three studies in that it established load control and progression through perceived exertion, evolving the volume and intensity of the training protocol, which may have made the applied protocol more robust (17).

Regarding quality of life, six studies evaluated this outcome using different tools. For the elderly population, only synchronous tele-exercise protocols were used. Synchronous tele-exercise appears to improve domains related to quality of life, including loneliness (15, 25). These findings may be linked to the tele-exercise protocol being conducted in groups and in real-time, which could foster social connections between students and instructors.

Regarding synchronous tele-exercise, individuals affected by multiple sclerosis (MS) demonstrated significant interaction effects on quality of life, whether through MS-specific questionnaires or general quality of life assessments. The studies used synchronous tele-exercise protocols, specifically tele-yoga and tele-pilates (22, 23). In a recent systematic review conducted by Sánchez-Lastra et al. (2019), the authors reported significant improvements in the quality of life of people with multiple sclerosis who practised Pilates, supporting the findings of this review (37). However, only three studies evaluated quality of life improvements through Pilates, and only two reported significant differences (38, 39). For yoga, a recent systematic review (40) did not find significant differences in quality-of-life improvements for yoga practitioners, contrasting with the findings here. These cited studies have distinct methodological approaches, which may lead to conflicting results. Further research, such as high-quality randomised clinical trials, is needed to better understand the effects of these practices on the MS population.

Regarding asynchronous tele-exercise, the authors did not observe significant differences in the quality of life for people with Parkinson's (18). Nonetheless, the sedentary subgroup experienced a clinically relevant improvement in quality of life with the asynchronous tele-exercise protocol. It seems that in sedentary populations, even small doses of physical activity can lead to a noticeable improvement in perceived quality of life. The levels of motor and cognitive impairment associated with Parkinson's may contribute to the differences observed within the subgroup. Current literature suggests that physical inactivity worsens the quality of life in this population, indicating that the more inactive an individual is, the lower their quality-of-life indicators (41). However, more research is needed on the impact of both asynchronous and synchronous tele-exercise for this population.

When evaluating functional capacity, synchronous tele-exercise showed interaction effects in elderly individuals assessed with the 6 Min Walk Test (TC6) (16), and moment effect for the 10-Meter Walk Test (TC10) (21). Asynchronous tele-exercise demonstrated a moment effect for the 5-Meter Walk Test (TC5) (8), while a mixed synchronous and asynchronous tele-exercise protocol achieved a moment effect for subjective assessments (26). Despite the heterogeneity of the programs, tele-exercise appears to have the potential to improve functional capacity in the elderly, yet only one study demonstrated a significant interaction effect (16), highlighting the need for more robust studies with this population. It is important to note that current research suggests functional capacity may be influenced by the type of physical exercise program used. The literature indicates that multimodal programs may be more effective in improving the functional capacity of the elderly, which could influence the results (42, 43).

Other three different population groups showed a moment effect for synchronous tele-exercise (19, 22) and asynchronous tele-exercise (18). However, it is important to note that half of the studies addressing functional capacity are either quasi-experimental or non-randomised. More research with robust methodologies is needed to better understand the effects of tele-exercise on functional capacity.

Regarding strength outcomes, both synchronous and asynchronous tele-exercise protocols focused on lower limb strength, which was reflected in the results. Of the nine studies that assessed strength, six evaluated lower limb strength, two assessed lower and upper limb strength, and one assessed only superior limb strength. Only synchronous tele-exercise protocols (15, 16) showed significant differences between groups with interactions effects for the elderly population, aligning with current literature on the benefits of strength training for older adults (44–46). Only one study used asynchronous tele-exercise protocols. Although it did not find a significant group effect, a significant moment effect was observed (8).

Synchronous and asynchronous tele-exercise appear promising for increasing lower limb strength in the elderly population, with synchronous tele-exercise showing better results in this group. Synchronous tele-exercise appears promising for strength gains in adults with Down syndrome, aligning with current findings on resistance training for this population (35). However, the study in question lacks a control group, which renders the strength of this finding fragile, as it may not truly represent an improvement attributable to the intervention. Controlled studies are necessary to clarify whether there is an interaction effect between groups.

Only isometric strength seems to have been benefited by tele-exercise protocols for upper body strength. Women with obesity and overweight and people with recent COVID-19 showed moment effects on strength, but only with small effect sizes (17, 28). Generally, tele-exercise is performed with low load, few implements, and a focus on upper body exercises, which may influence the results regarding strength gains, particularly for upper body strength.

Finally, synchronous and asynchronous tele-exercise protocols appear to be effective regarding pain outcomes. Asynchronous tele-exercise showed significant improvement in reducing lower back pain with a large effect size in sedentary, obese, and overweight pregnant women (7). These findings align with current guidelines for pregnant women (47, 48), where structured physical exercise can help manage and reduce lower back pain. However, the study by Bernardo et al. (2024) was the only study that used asynchronous tele-exercise for the pain outcome (7).

Other studies evaluated pain with synchronous and mixed tele-exercise protocols. For people with multiple sclerosis, both tele-yoga and tele-pilates showed significant interactions effects improvements. Pilates and yoga, in their in-person form, are commonly used for pain management (49–52), and synchronous tele-exercise appears to provide similar benefits for people with multiple sclerosis. Synchronous tele-exercise also showed greater improvements in pain scales compared to asynchronous methods in adults during lockdown without specific pain complaints. However, both types of protocols reduced average perceived pain scores. The literature does not have a consensus on which type of exercise is most beneficial for pain, whether chronic, acute, or neuropathic. However, it is well-established that well-managed physical exercise is a crucial ally in reducing pain and improving the quality of life across various populations experiencing different types of pain (53). This seems to be reflected in the tele-exercise protocols. Currently, the methodological quality of studies on tele-exercise is low, and the heterogeneity of populations makes a robust analysis even more challenging. Nonetheless, tele-exercise emerges as a promising approach, particularly synchronous tele-exercise, which demonstrates more robust results and, typically, outcomes similar to those of in-person exercise when compared within the same population group. Thus, it offers a potential way to include various populations in physical exercise practices, particularly those with limited or no access to exercise environments. More studies with high methodological quality are needed to make a clearer view of the effects and limitations of tele-exercise.

## Data Availability

The original contributions presented in the study are included in the article/Supplementary Material, further inquiries can be directed to the corresponding author.
